# Place Matters: A Study on the Influence of Birthplace and the Place of Development on Soccer Academy Players’ Careers

**DOI:** 10.3390/sports12040099

**Published:** 2024-03-31

**Authors:** Lander Hernández-Simal, Julio Calleja-González, Jon Larruskain, Alberto Lorenzo Calvo, Maite Aurrekoetxea-Casaus

**Affiliations:** 1 Faculty of Education and Sport, University of Deusto, 48007 Bilbao, Spain; 2 Faculty of Education and Sport, University of Basque Country (UPV/EHU), 01007 Vitoria, Spain; julio.calleja.gonzalez@gmail.es; 3 Faculty of Kinesiology, University of Zagreb, 10000 Zagreb, Croatia; 4 Medical Services, Athletic Club, 48196 Lezama, Spain; j.larruskain@athletic-club.eus; 5 Faculty of Physical Activity and Sport Sciences-INEF, Universidad Politécnica de Madrid, 28040 Madrid, Spain; alberto.lorenzo@upm.es; 6 Faculty of Social Sciences and Humanities, University of Deusto, 48007 Bilbao, Spain; maurreko@deusto.es

**Keywords:** soccer, birthplace effect, talent development, sporting growth, geographical mobility, talent selection, talent promotion

## Abstract

The effect of birthplace (the place where a player is born and grows up) is one of the key variables associated with soccer player development and achievement. However, recent studies have questioned the influence of contextual variables on selection and promotion processes related to birthplace. The main purposes of this study were: (1) establish whether there is a difference between the birthplace and sporting growth according to the stages of entry into the academy, (2) to evaluate the influence of geographical and contextual variables on sporting development, and (3) assess the chances of making a professional team debut. Applied logistic regression was used in order to analyse the birthplace and growth of 1411 male soccer players, of which 40.1% are incorporated in the U-12 category from a Spanish First division club, and the results showed a statistically significant association between a change from one’s birthplace to the place of sporting growth and later success (79%). Key contextual variables such as number of inhabitants, population density, proximity to sports facilities, average household income and presence of sports clubs have been considered. The analysis of the contextual variables revealed that there was a positive relationship between certain variables, like a higher population, and being selected for the academy (*p* < 0.01; r = 0.28). Finally, the findings showed that players who experienced a geographical transition were 38% more likely to make a professional debut. The importance of considering the place of sporting growth when analysing the “birthplace effect” has therefore been demonstrated.

## 1. Introduction

The globalisation of soccer and its growing popularity in recent decades have established it as one of the most competitive and influential sports throughout the world [1]. This phenomenon has significantly transformed talent management in soccer [2], which focuses intensively on every stage of the developmental process, from the initial identification and selection of promising players to their growth and subsequent promotion into the ranks of elite soccer [3,4,5]. In this dynamic context, the academies of professional clubs have acquired transcendental importance [6]. Specifically, these professional academies play a vital role in the present and future sporting development of players [7,8,9] and are responsible for managing the complex processes of talent management.

The outcome of actual talent emerges from the interaction between innate, acquired, and contextual factors [10]. Innate factors include inherited qualities that lay the foundation for the subsequent development of sporting talent, such as genetics, physical constitution, and cognitive abilities, among others [11]. Acquired factors, in contrast, are developed through experience, training, and one’s environment [4]. Finally, contextual factors, such as birthplace, population size, population density, sports infrastructure, family, and socioeconomic conditions [12,13], are just some of the conditioning factors that can influence talent management processes [13].

Among all these contextual factors, geography is becoming increasingly relevant, not only in terms of a player’s specific place of birth but also because of the opportunities and experiences that such a place can provide in the sporting growth of the soccer player [14]. In some of the first studies on this topic, the variables most examined by scientific researchers were those related to the population and the configuration of the territory [15], including the size and density of the population of the players’ birthplaces [16]. However, recent studies have addressed other variables, such as the proximity to and number of clubs [17], the role of the socioeconomic status of families [18], and the quantity and quality of the available facilities [19].

The process of identifying players’ birthplaces initially appears simple; however, some inconsistencies have emerged [20]. Recent research [21] underlines the need to further refine this starting point by adapting it more precisely to the player’s sporting growth environment, which does not always coincide with the place of birth [13]. While for many athletes, their place of birth and place of formative development are identical, others are born in one community and, for various reasons, move to another during their formative years [16,22,23]. Therefore, within the context of place of birth, an element of uncertainty arises that makes it difficult to understand this variable as a determining factor. This uncertainty lies in the conceptual controversy between place of birth and place of athletic growth, an issue that Ishigami [20] highlighted as a limitation of his own study. Therefore, further exploration into the effect of place of birth requires that we look not only at place of birth but also at the place of athletic growth and that we study the impact of the variables relevant to this geographical transition [24,25,26]. Therefore, the main aim is to establish whether there is a difference between the birthplace and sporting growth according to the stages of incorporation of the players, which is the guiding question of this research.

In an attempt to establish the relationship between birthplace and sport growth, a second aim is to examine the differences between the number of contextual variables associated with birth municipalities and those of sport growth [13]. Throughout the specialised literature, considerable attention has been devoted to the study of contextual factors, among which community size and population density have been the most studied [17,27,28,29]. These variables have been the subject of extensive research due to their potential influence on athletes, and their study has revealed important connections between the socio-demographic environment and the progress of players in sports [13,20,23,26,30].

Although early studies on the influence of birthplace on the likelihood of entering professional sports focused on players born in cities with less than 1,000,000 inhabitants [27], later research highlighted how the chances of becoming an elite athlete were lower in both very small and very large communities [23,30]. In particular, authors such as Rossing [17] have emphasised the greater likelihood of achieving professional status by being born in higher-density locations. More recent studies suggest that an increase in density increases the probability of achieving professional status in soccer [28]. However, the density variable betrays some inconsistencies, as several studies [29] have reported higher soccer participation rates in smaller urban settings. In line with this finding, Teoldo and Cardoso have argued that there is greater potential in small- and medium-sized cities for talent identification and development processes [15]. In this vein, other studies have indicated that athletes born in small- or medium-sized cities were more likely than others to reach the highest level in their sports [16].

In addition to population size, other studies have highlighted the relevance of the geographical proximity of youth players to high-performance clubs [17]. This could be because proximity provides them with access to superior resources, such as high-quality coaching, excellent facilities, and participation in high-level competitions [24]. A greater number of clubs can contribute to the identification and development of sporting talent, as well as to the broader promotion of physical activity and sport in society [29]. In this sense, studies have revealed how these geographical contextual variables can influence the likelihood of becoming elite players [31].

The purpose of this study is to evaluate how birthplace and athletic growth influence the trajectories of players, with the aim of better understanding the dynamics that affect their chances of selection and success in professional soccer. This research seeks not only to identify significant patterns between contextual factors and sports achievements but also intends to contribute to the existing scientific literature by providing a new perspective of analysis through the concept of players ‘sporting growth’. From an academic standpoint, the study broadens the understanding of geographical variables in athletic development, in contrast to the usual parameters of talent management, which focuses on the innate factors of the athlete and relegates the geographical factor. 

In practical terms, the findings could serve as a guide for coaches, talent academies, sports organisations, and policymakers to design more effective strategies in talent detection and development, taking into account the importance of the environment. Moreover, this analysis could have significant implications for resource planning and the implementation of support programs aimed at maximising the potential of soccer players from an early age.

## 2. Materials and Methods

### 2.1. Design

A single evaluation of a set of different variables was carried out. The data set contained the data of soccer players in which a group of variables related to certain sporting qualities of the player were collected, such as their playing position and their positionality, which were not used for this study. In addition to their birthdate were variables that provided information on the incorporation of the player into the club, such as the club of origin, the stage of incorporation, the number of years in the club and the number of different clubs in which he participated. To this group were added a series of variables that helped to provide information on the context of the birthplace and municipality of sporting growth, such as the number of inhabitants, the density of the municipality, the distance to the club, the number of clubs or family income in the municipality.

### 2.2. Participants

The selected sample consisted of a total of 1411 male soccer players belonging to the last 30 seasons of a Spanish first-division professional soccer club whose working philosophy was based on the youth academy (from 1992 to 2021). The stages at which the players were incorporated into the academy and the players who had managed to make their debut on the club’s professional team (playing in an official club match) were compiled into a dataset. Of the total of 1411 players, the average number of years at the club is 5.07 (SD 3.80), although the average number of years as a soccer player stood at 9.80 years (SD 7.331). These players have been through an average of 3.63 teams before being incorporated into some of the categories. Although the highest percentage of players joined in the U-12 category (40.11%), it is noteworthy that there is a strong contingent of players joining in the U-16 category (15.1%) and U-18 category (18.5%), accounting for 33.6% of the total number of players.

All records correspond to players who participated in at least one season (with a valid date from the Spanish Football Federation). The players selected for the study had a minimum chronological age of 9 years and a maximum age of 35 years. Players who were on probation in the academic selection process were excluded. All players who did not belong to the club development network linked to the main club were excluded.

### 2.3. Procedure

The data sample was provided by the club, who gave permission to utilise the data on the basis of an agreement for the elaboration of the study and its publication (data release agreement signed on 28 April 2022). After approval, the objectives and characteristics of the research were explained to the general manager of the Club’s Data Department. The club’s policy states that the data collected on the players is subject to the data release protocol as it is necessary in the system of management and assessment of the players’ sporting talent. Both the players and their managers are aware of this transfer. Apart from this procedure, this project is coordinated within the Deusto Sports and Society Team of the University of Deusto, an entity that requires all its research teams to comply with the ethical principles of the Declaration of Helsinki [32] (2013). This research had the approval of the ethics committee of the University of Deusto Research Ethics Committee reference: ETK-3/21-22. 

### 2.4. Statistical Analysis

Detailed data were collected on the players’ birthplaces, as well as the municipalities in which they developed their sporting careers. The information was obtained through an exhaustive analysis of the records and databases provided by the club. The confidentiality and anonymity of the data were guaranteed to preserve the privacy of the players involved in the study (i.e., by coding each of the players who participated).

The contextual variables considered were those related to the region’s number of inhabitants, its density, the distance to the location of the high-performance centre, the economic situation of the families, and the number of clubs (Table 1).

The frequencies of the selected players were extracted by stages of incorporation, beginning with the players who entered the first team of the academy structure (U-12) and extending to those who directly entered their first professional teams (debutants). All the birthplaces of the players were collected (through the registration of their identity cards), and the places of sporting growth were determined separately. Players who developed in the same place where they were born were called “players of constant sporting growth”, and those who moved to a different place of development after birth were called “players of rooted growth” (Table 2).

Data were presented as means and standard deviations. The Kolmogorov–Smirnov test was used to assess normality (n > 50). After testing for normality, parametric and non-parametric tools were used. These are described below, in line with the objectives.

#### 2.4.1. First Objective

Chi-square tests were conducted to determine the association between players with the same birthplaces and places of growth (“players of constant sporting growth”) and those with different places of birth and sporting growth (“player of rooted growth”) (Table 2).

#### 2.4.2. Second Objective

**Figure 1:**
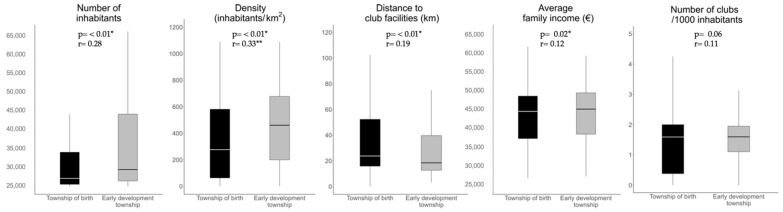
Distribution and dispersion of data for each contextual variable with respect to place of birth and sporting growth. * Moderate significance ** Highest significance.

In the analysis of the contextual variables, *p*-values and R-values were calculated to evaluate both the statistical significance and the effect size associated with each variable. Significant *p*-values were considered to be equal to or less than 0.05. Moderate effect values were between 0.3 and 0.5, while those above 0.5 were considered highly significant. Values below 0.3 were considered indicative of a small effect [33]. The significance and effect size results for each contextual variable are presented together with boxplots in Figure 1. The change in municipalities between birthplace and place of sporting growth was specifically addressed by considering the median and interquartile range (IQR) values for all variables.

#### 2.4.3. Third Objective

To assess the probabilistic indices of a professional team debut, odds ratios (OR) and 95% confidence intervals (CI) were calculated by comparing the relative frequencies of birthplaces and places of sporting growth between players who changed their municipalities and those who did not. Subsequently, a hierarchical binary logistic regression model was constructed to determine the probability of a player making a debut on a professional team (Table 3).

Statistical analyses were performed using R Studio software, an integrated development environment (IDE) for the R programming language. This program facilitated the execution of significance tests, OR calculations, and the construction of hierarchical binary logistic regression models. A *p*-value of less than 0.05 was considered significant.

## 3. Results

### 3.1. First Objective

Table 2 presents the analysis of the frequencies by stage of academy entry among players who switched from their birthplace to a new place of sporting growth, compared to those who did not make the transition.

The results of the chi-square test revealed a statistically significant association between the change from one’s birthplace to a new place of sporting growth, with 79% certainty (observed chi-square value of 11.070% and calculated chi-square value of 63.77%).

In the U-12 category, 210 players (37.1%) experienced a change in municipality from their birthplace compared to a new place of sporting growth, while 356 players (62.9%) maintained the same municipality between birth and growth. At the U-14 level, this trend was maintained, with 61 players (40.7%) who changed and 89 players (59.3%) who did not. Likewise, in the U-16 category, 95 players (44.4%) changed, and 119 players (55.6%) stayed in their places of origin. The U-18 category showed a similar distribution, with 125 players (47.8%) switching and 137 players (52.2%) remaining in the same place. On the second team, 107 players (62.6%) changed their place of birth compared to where they grew up, while 64 players (37.4%) stayed in the same place. Finally, on the first professional team, 38 players (79.2%) changed to a new place of development, while 10 players (20.8%) did not make such a change.

### 3.2. Second Objective

The analysis of the contextual variables of sporting development is reflected in Figure 1. In terms of the variables of the number of inhabitants, density, distance, and income, the values were significant (*p* < 0.5), while the number of clubs had non-significant values (*p* > 0.5). Only density had an r-value with moderate values (r = 0.33). Population size, represented by the number of inhabitants, revealed a significant correlation with player selection (r = 28), with *p* < 0.01. Furthermore, distance to the academy facilities was revealed to have values of r = 0.19 and *p* < 0.01. Accordingly, the results conclusively support the existence of a statistically significant connection between these two factors (i.e., change in residence and distance). 

Overall, the results of the variables show that the higher the number of inhabitants, the higher the population density, the shorter the distance to the club’s high-performance centre, and that there is a positive relationship between these variables and being selected for the academy.

### 3.3. Third Objective

Table 3 presents the OR results for assessing the chances of making a professional team debut in relation to “players of constant sporting growth” and “players of rooted growth” at the different stages of academy selection. With regard to the data presented, first-team players have been excluded from this analysis. 

In this group, the incorporation of players signed directly from other teams without going through the different categories generates a distorting effect on the understanding of the sporting growth variable in the selection processes, and for this reason, they have not been included in the analysis. 

For the full interpretation of the table of players who have been promoted, those who have played at least one official match with the professional team have been taken into account (transfer signing).

In the U-12, U-14, U-16, U-18, and U-23 entry stages, no statistically significant association between the change in municipality and the chances of making a debut in the club’s professional team emerged. However, when consolidating the data from all entry stages, including the professional team, a different trend was revealed (1.38). 

When we analysed the data by stage, we found that the municipality of birth and growth, whether constant or different, had no relationship with the chances of a player selected for the academy making it to the professional team.

Conversely, looking at the overall numbers, the OR of 1.38 (95% CI: 1.04–1.84) indicated that players who switched municipalities were approximately 38% more likely to debut compared to those who did not switch, and this increase was statistically significant. Therefore, a higher proportion of players with different municipalities of birth and growth was observed in those who had made it to the first team from the different stages of incorporation or who were directly selected for the first team (50% “rooted”, 113/113), compared to those who did not make the first team (42% “rooted”, 497/687).

## 4. Discussion

This study set out to achieve three objectives: first, to analyse the effect of place of birth on sporting growth; second, to evaluate the influence of geographical and contextual variables on sporting growth; and finally, to study the phases of selection and promotion based on birth and sporting growth. The main results obtained demonstrated the existence of a statistically significant association between the change in birthplace to a new place of sporting growth, and that the greater the number of inhabitants, the greater the population density, and the shorter the distance to the high-performance centre, and thus that there was a positive relationship between these variables and being selected for the academy. Finally, players who experienced a geographical transition were more likely to make their debuts on the professional team.

### 4.1. First Objective: To Analyse the Effect of Place of Birth as a Function of Sporting Growth

The results showed in a statistically significant way that the change in the players’ birthplace to a new place of sporting growth was substantial at all stages of incorporation. The conventional notion that the place of birth should be the starting point for the study of the “birthplace effect” phenomenon has thus been challenged [20,24] based on the reality observed, as in many cases, these two places can be markedly different, as the results of the present study show.

Although this trend was maintained at all stages of incorporation, it became more significant as the age of the players increased, with the most representative group being those players who joined the first team directly (79.2%). At the same time, players who joined the U-12 stage represented the lowest percentage in this geographical transition (37.1%). The results obtained in the study, which reveal a low incidence of changes in municipality between the birthplace and place of sporting growth in the early years of play (U-12), can be understood through various dynamics that characterise the initial phases of sports training. In these early stages, the influence of family and community appears to be a preponderant factor [34]. Decisions about place of residence are influenced by considerations beyond the purely sporting sphere, such as parents’ job opportunities, proximity to relatives, and general quality of life [35]. In this sense, children’s geographical mobility may be constrained by these non-sporting variables. Logistical constraints may also play an important role [36]. For instance, dependence on parents or guardians for transport, coupled with the distance among municipalities, may make it difficult for children to travel beyond the boundaries of their birthplaces in search of sporting opportunities [37,38].

School stability may also present itself as another determining factor. Resistance to significant changes in children’s education may influence the decision to stay in the same municipality [39,40,41]. In addition, the lack of pronounced sport specialisation at these early ages could mean that children participate in local sports programmes without the need to explore other facilities or specific programmes elsewhere, with the local sports offerings considered sufficient at these early stages, thereby eliminating the perceived need to seek sporting alternatives elsewhere [42,43]. In contrast, a detailed analysis of the data reveals that as the level at which players enter increases, the percentage of players who change their place of birth relative to their place of growth also changes [44].

This phenomenon raises the question of why this increased mobility occurs at this stage compared to the early stages of sporting growth. One possible explanation is the intensification of competition and sporting opportunities as players progress into higher age groups [45]. At these stages, the pressure to excel and access more advanced developmental programmes may motivate players and their families to seek out sporting environments that are more conducive to sporting growth and visibility [46]. In addition, as players advance in their sporting careers, they are likely to become more autonomous in their decision-making and actively seek out environments that offer them better opportunities for competitive sports development [47]. The influence of coaches and the quality of training programmes may also be determining factors in the decision to change one’s municipality in search of more specialised training [48]. In this sense, the attention of professional scouts and clubs could also be a key influence [49,50]. In addition to the above, at older ages, the search for talent intensifies, and outstanding players may receive offers to join academies or clubs in different geographical locations, thereby driving a major geographical transition [7,51,52]. Specifically, this could motivate families to consider relocating to places that offer better sporting development opportunities and exposure to higher levels of competition.

In parallel with these external factors and in relation to internal factors, the biological and psychological maturation of players also plays a crucial role [53,54]. As young athletes reach maturity, they not only become physically more capable of facing more demanding challenges but also develop greater self-management in making strategic career decisions [52]. This phenomenon, known as “mature age talent identification”, implies that sports talents are often identified and selected at a more mature age, wherein the player’s skills, attitudes, and physical abilities may have reached a level of development that allows them to excel and adapt quickly to more competitive environments. The interaction between individual maturity and the selection of mature talent may become a dynamic factor in mobility and relocation decisions, which suggests an intrinsic link between the evolution of sporting careers and the progression in the age and maturity of players [55].

The most significant representation is when players are selected directly by the professional team of the structure, which can be attributed to a variety of reasons. One of the most prominent reasons may be the pursuit of opportunities and exposure to a higher level of competition [56]. As players progress to the professional level, competition becomes more intense, and opportunities to excel and be recruited by elite teams may be found only in specific geographic locations [57]. Players and agents may make the strategic decision to seek out sporting environments that maximise the chances of visibility and career progression. In this regard, the influence of sports agents may also be a key factor [58]. Players’ agents and representatives often play a crucial role in transfers and transfer negotiations, even in amateur soccer and may actively seek opportunities for their teammates on various teams [59,60].

### 4.2. Second Objective: To Assess Geographical Contextual Variables in Sport Development

The second objective of this study was to assess the differences among the contextual variables between the territories involved in the geographical transitions of players. 

It is essential to recognise that the transition of players from their place of birth to their place of sporting growth does not go unnoticed [16]. On the contrary, it is fundamentally influenced by the particularities of the environments of origin and destination. These particularities can encompass a broad range of factors, including, but not limited to, population size, density, socioeconomic status, sports supply and infrastructure, and professional development opportunities, among others [17,61,62].

In this sense, and sticking to the variables of our study, it can be observed that the data referring to population size [29], density [63] and the distance to a high-performance centre [17] were the most significant contextual variables indicative of the changes that occur within the territories when transitioning from one’s birthplace to a new place of sporting growth [61].

The mobility of players to larger and densely populated municipalities may be directly linked to the pursuit of higher-potential sporting opportunities and resources [13]. In urban or metropolitan areas, the concentration of quality facilities, advanced development programmes, and more intense competition may be particularly attractive to players and their families who aspire to reach higher levels of sport [28]. These environments offer potential access to a more extensive network of clubs, academies, and specialised coaches, thereby creating an ecosystem conducive to sporting growth and visibility [23]. Furthermore, population density and the resulting formation of more dynamic sporting communities facilitate regular interactions with fellow players and participation in more competitive leagues, which exposes players to a greater diversity of styles of play that could contribute to their all-round development [64,65,66].

Adding to this dynamic, the concept of “performance level” takes on particular relevance. It has been postulated that a higher level of sporting demand, characteristic of more competitive environments, not only improves technical and tactical skills but also strengthens the psychological resources of athletes [67]. According to Collins and McNamara’s research, the process of facing and overcoming minor difficulties or “traumas” on the road to success is an integral component in the formation of a resilient athlete [68]. This “rocky road to the top” suggests that talent requires such challenges to cultivate tenacity and resilience in the face of future setbacks. Therefore, players who experience an environment with a high level of performance may be better prepared to handle and overcome adversity, which gives them an advantage in their long-term athletic development [69].

In addition, geographic proximity may translate into more direct access to opportunities, allowing players to participate more actively in training programmes and events related to high-performance clubs [17]. This physical proximity may facilitate greater interaction with the sporting environment and potentially have a positive impact on player skill development [70]. This distance can also have a cultural influence by ensuring greater identification with the soccer community, which can also facilitate a closer connection to the local soccer culture. This cultural transition also means that significant cultural boundaries can be crossed in the process of adapting to the new value systems, social norms, and lifestyles prevalent in the host club environments [71,72]. This cultural transition is an issue that has received considerable attention in soccer settings, as it has been recognised that acclimatisation to a new sporting and social culture can have a substantial impact on player well-being and performance in the field [73,74].

### 4.3. Third Objective: Selection and Promotion Based on Birth and Sport Development

The third objective of this study was to explore the complex dynamics between the geographical transition of players from their place of birth to the location of their growth in the sport and its impact on the selection and promotion phases within the club. 

Although the results are inconclusive, the findings indicate that there is no direct or deterministic correlation between whether players maintain their initial residence or change this location and their chances of promotion to the first team. However, an intriguing trend was revealed, which showed that a higher proportion of players reaching the first team tended to have a history of changing their place of sporting development, with 50% reporting different municipalities of birth and development. Future studies should explore this issue further.

These data could be interpreted as an indication that players who are willing to make significant geographical changes may be more open to new experiences and challenges, or that mobility may be a reflection of their commitment and determination to reach higher levels in soccer [75,76]. Furthermore, the fact that this phenomenon is more pronounced in the U-18 category is especially revealing. Specifically, this finding could be linked to the concept of mature-age talent detection, which supports the idea that players should be selected once they have completed their maturational processes [55].

At the threshold of the U-18 category, players are not only reaching their peak in terms of physical and technical development but also acquiring the psychological and emotional maturity that allows them to better handle the pressure and expectations of high performance [76]. Selection at this mature stage could be beneficial for both clubs and players, as detected talents are more likely to be prepared for the demands of performance and to have a clearer and more experienced view of the competitive environment [77,78].

Overall, these findings suggest that clubs could consider adaptability and life experience as important factors in their selection and promotion decisions, thereby emphasising not only technical skills but also resilience and the ability of players to adapt to new environments and challenges. These latter features may very well be fundamental determinants of success at the highest levels of professional soccer [79,80,81,82,83].

## 5. Conclusions

### 5.1. First Objective

The geographical transition from one’s birthplace to a place of sporting growth is a common and significant phenomenon in the development of soccer players.

### 5.2. Second Objective

The contextual variables of population size, density, and, to a lesser extent, distance to a club’s sports centre play a key role in the geographic transition of players. The preference for municipalities with higher populations and increased density during sporting growth suggests a tendency to seek environments that offer greater opportunities and resources for soccer development, as well as a higher level of demand.

### 5.3. Third Objective

On the one hand, the differences or similarities between the municipalities of birth and growth had no relationship with the chances of a player being selected for the academy reaching the first team. On the other hand, a higher proportion of players with different municipalities of birth and development was observed in those who had come to play on the first team, whether from the academy or directly, compared to those who had not.

With regard to the strengths of the study, the following are highlighted: first, incorporating both the place of sporting growth and birthplace as joint variables for the identification of players in academies marks a milestone in studies on the identification and selection of talent in professional academies. Thus far, the academic literature has focused exclusively on the birthplace when explaining the contextual factors relevant to talent management processes.

Second, this study stands out for its innovative approach to addressing the complexity of sporting development in soccer players, thereby providing a deeper and more complete understanding of the process of the “birthplace effect” phenomenon.

Finally, the breadth of data spanning multiple stages of incorporation allows for a more complete analysis of how a geographic transition can influence different moments in the sporting development of young players, thus providing further insight into what happens at each stage of growth.

Overall, this study not only challenges conventional notions about the impact of “place of birth”, but also expands our overall understanding of how geography influences sport development from selection to promotion. As such, it provides valuable contributions to the existing literature.

Regarding practical applications, the identification of two distinct profiles, “consistent growth players” and “rooted growth players”, suggests new perspectives on how and when players make such a geographic transition and its impact on their development. Nevertheless, detailed monitoring of players is required to understand the spaces or places through which their sporting growth takes place and the conditions that these places bring to the development of sporting talent. Final del formulario

In terms of limitations, variability in the quality and availability of birth and sport growth data may have introduced potential biases. Specifically, reliance on historical records and a possible lack of uniformity in the data collection could affect the accuracy and completeness of the data. In addition, the exclusion of more specific contextual factors, such as local sporting infrastructure, could have limited the ability of the study to address all the influential dimensions of sports development.

Another limitation lies in the lack of direct control over individual variables, such as the type of training received or the quality of the sporting growth clubs. This could make it difficult to attribute specific results to particular causes.

Finally, generalisation of the findings to sporting contexts outside of soccer or to other geographical regions should be approached with caution, as the dynamics of each context may vary significantly. These results refer to a unique aspect of the world that is difficult to replicate in other realities.

## Figures and Tables

**Table 1:** Definitions and descriptive data for contextual variables.

Geographical and Contextual Variables	Unit of Measurement	M	SD	A
Inhabitants	Number of inhabitants.	10,215.38	34,791.128	8238
Density	Population living in the municipality per km^2^.	1787.44	1645.48	711
Proximity	Kilometres (km^2^) of the municipality with a high-performance centre.	39,351	46,503	2556
Household income	Total household income.	44,235.42	7799.79	1056
Clubs	Number of soccer clubs in the municipality.	1403	1639	1174

M: Represents the mean; SD: standard deviation; A: skewness of the data presented.

**Table 2:** Difference between birthplace and place of sporting growth according to stages of incorporation into the academy.

Incorporation Stage	Total % (Abs)	Players of Constant Sporting Growth Birthplace vs. Sporting Growth % (Abs)	Player of Rooted Growth Birthplace vs. Sporting Growth % (Abs)	% Different Birthplace vs. Sporting Growth
U-12	40.1 (566)	37.1 (210)	62.9 (356)	37.1
U-14	10.6 (150)	40.7 (61)	59.3 (89)	40.7
U-16	15.1 (214)	44.4 (95)	55.6 (119)	44.4
U-18	18.5 (262)	47.8 (125)	52.2 (137)	47.8
U-23	12.1 (171)	62.6 (107)	37.4 (64)	62.6
First team	3.4 (48)	79.2 (38)	20.8 10	79.2

Players of constant sporting growth: Players who had changed their birthplace to a different place of sporting growth. Players of rooted growth: Players who maintained the same place of birth and sporting growth. % Different: Percentage of players whose place of birth differs from their place of sporting development.

**Table 3:** Possibilities of debuts between players with the same or different municipalities of birth and growth in the selection phase.

Incorporation Stage	Player of Rooted Growth No Debut	Players Constant Growth No Debut	Player of Rooted Growth Yes Debut	Players Constant Growth Yes Debut	OR (95% CI)
U-12	187	345	11	23	0.88 (0.42–1.84)
U-14	44	68	15	23	1.01 (0.48–2.14)
U-16	118	130	6	8	0.83 (0.28–2.46)
U-18	62	93	29	30	1.45 (0.79–2.65)
U-23	86	51	15	19	0.47 (0.22–1.01)
Total	497	687	76	103	1.38 (1.04–1.84)

## Data Availability

The data are not publicly available due to privacy and ethical restrictions. The data are not publicly available due to containing information that could compromise the privacy of research participants.
